# A fine construction method of urban road DEM considering road morphological characteristics

**DOI:** 10.1038/s41598-022-19349-4

**Published:** 2022-09-02

**Authors:** Yu Tao, Lei Tian, Chun Wang, Wen Dai, Yan Xu

**Affiliations:** 1 School of Geographic Information and Tourism, Chuzhou University, Chuzhou, 239000 Anhui China; 2 College of Forestry, Nanjing Forestry University, Nanjing, 210037 Jiangsu China; 3 Anhui Province Key Laboratory of Physical Geographical Environment, Chuzhou, 239000 Anhui China; 4 School of Geographical Sciences, Nanjing University of Information Science and Technology, Nanjing, 210044 Jiangsu China

**Keywords:** Environmental sciences, Solid Earth sciences, Engineering

## Abstract

Urban road DEM is not only an important basic geographic information data of the city, but also an important element to describe and express the urban topography, and it is an indispensable part of the construction of the smart digital city, urban planning and urban surface process simulation. Previous methods for constructing urban road DEMs do not sufficiently consider the actual morphological characteristics of urban roads, and morphological distortion is evident in the expression of urban roads, seriously affecting the application of urban rainfall flood simulation and urban pipe network design. In response to these problems, this study proposed a considering morphological characteristics fine (CMCF) method of urban road DEM construction, selected a typical urban road area in the Jianye District of Nanjing City in China as the study area, used the 1:500 digital line graphic as data source, hierarchized roads in accordance with different morphological characteristics from the perspective of DEM construction, constructed the corresponding DEMs, and finally merged all levels of road DEMs to produce a complete high-precision urban road DEM. Results showed that the DEM constructed using the CMCF method not only exhibited higher elevation accuracy than the urban road DEM constructed using previous methods, i.e., inverse distance weighting (IDW) and triangulated irregular network (TIN) methods, with a mean error and a root-mean-square error of 0.015 and 0.060, respectively, but it can also accurately express the spatial element composition form and road morphological characteristics of urban roads, avoiding the distorted expression of road morphological characteristics. This study can provide a reference for a new DEM construction method and data support for smart digital city construction and urban surface simulation.

## Introduction

As one of the most important national basic geographic information, a digital elevation model (DEM) is a digital expression of surface morphology, and it contains a huge amount of topographical and geomorphological information necessary for the analysis of geographic and non-topographic characteristic applications^1^. As a political, economic, and cultural center on a regional scale, a city is an advanced pattern of human settlement. High-precision urban DEM is important geographic information for cities; it cannot only provide abundant topographic data for establishing digital twin cities^2,3^, but it also plays an important role in urban hydrological system simulation^4^, natural disaster warning^5^, and environmental science protection^6^. As typical artificially transformed terrain, urban roads carry the basic skeleton of urban terrain morphology. They are also important to control lines of urban terrain strip compartment, and a key element for expressing digital city and urban surface process simulation. Therefore, the construction of urban road DEM is the key step in urban terrain modeling, and how to quickly and accurately construct urban road DEM is worthy of in-depth exploration^7^.

At present, research on DEM construction focuses on data sources that adopt a single data source or multiple sources for constructing DEM. With the development of modern Earth observation technologies, LiDAR^8–10^, interferometric synthetic aperture radar (InSAR)^11–13^, and unmanned aerial vehicle (UAV)^14–17^ have provided a huge amount of data for the construction of DEM^2^. However, the process of adopting such data to construct DEM is inevitably influenced by the limitations of these data themselves. For example, the accuracy of LiDAR data is extremely high at the center region of a road, but it significantly worsens on both sides of the road; LiDAR data are also expensive and their acquisition is slow, and correspondingly, data processing is costly and data release cycle is slow^9,18^. InSAR technology can provide massive, fast, high-precision, high-resolution, and real-time surface data for the construction of DEM in cloudy and foggy areas where traditional surveying is difficult to perform^12,13,19,20^. However, the urban terrain is complex, and laying control points is difficult. Moreover, the outer boundaries of urban roads typically cannot obtain actual surface information due to the overlapping shadows of trees and vegetation, leading to the lack of road elevation information in InSAR data. Despite certain results of adopting UAV-generated digital surface model point cloud data to construct high-precision DEMs^21–23^, obtaining accurate urban road ground and boundary information is more difficult due to the interference of irrelevant information, such as street tree shading and road vehicles. Thus, the application of UAV point cloud data to the construction of refine urban road DEMs requires further studies.

The measurability of 3D oblique photography models provides the possibility of urban road information acquisition^24–27^; however, the absolute accuracy of such models must be further studied before they can be applied to large-scale high-precision urban DEM construction. Although the development of new Earth observation techniques has reformed DEM construction development to a certain extent, deficiencies still exist in urban road DEM construction. Large-scale digital line graphic (DLG) data, which are the basis for the construction of each city, exhibit the advantages of high accuracy, low cost, and large area. Compared with other types of data, DLG data can guarantee data accuracy based on cost conservation; they are among the better sources of urban DEM data at present^28–30^.

In high-precision urban DEMs, roads are the core feature elements that control the topography and constrain the elements of urban surface morphology. Urban roads effectively divide a city into individual topographic units, and they can be used as dividing and converging boundaries of individual parcel morphology in DEM construction^31^. Road DEM is not only a description of the geometric characteristics of artificial terrain, but it is also a representation of its semantic, relational, and process characteristics. Road DEM simplifies and abstracts complex road entities and transforms the study of physical roads into the study of road DEMs. In a traditional control photography survey, external control points are the key geometric positioning reference data. However, the acquisition of these data is limited by various factors, such as environmental factors of the acquisition site, climatic conditions, and human factors. The geospatial information of a high-precision urban road DEM can be used as high-precision geometric control information instead of traditional external control points, significantly improving the efficiency of urban road external data acquisition and processing.

As early as 1914, British researcher William Rees Jefferys proposed the idea that roads should be classified rationally in his article “The Secretary of the Road Board.” In recent years, many domestic and international researchers have studied urban road classification (road hierarchies) from different perspectives. For example, Cavar et al.^32^ proposed a method for classifying urban roads on the basis of Global Positioning System vehicle trajectories and urban infrastructure feature data. Liu et al.^33^ developed an urban road classification system that integrates “mobility class, street activity type, and travel mode priority” in a people-oriented manner. Sun^34^ presented a classification method for urban traffic planning roads from the perspectives of road function, traffic capacity, and service level, with road carrying capacity and throughput as the objectives. China’s urban road classification concept originated from Germany in the 1940s, and the country has established a relatively diverse classification system after years of development. Li et al.^35^ analyzed the historical evolution of China’s urban road classification process for urban road functions. They studied the classification of China’s urban roads by referring to the characteristics and principles of foreign road classification methods. Sun et al.^36^ constructed an urban road classification system that fully meets the needs of road landscape design by combining traditional road classification methods from a landscape perspective. Liu et al.^37^ proposed a 3D road classification system based on the improved domestic and international road classification system; their system exhibits “level with priority, function with classification, and mode with priority”. Yang et al.^7^ proposed an urban terrain element classification and expression method that uses the object-oriented approach for urban terrain modeling. Xie^38^ focused on the essence that roads serve people and presented a classification method for urban roads based on the principle of public transportation priority. However, all these urban road classification methods regard a road as a whole and use only a single mathematical surface for constructing DEMs^28,30^; hence, consideration of road morphology characteristics is lacking. Real urban roads are difficult to be expressed using a single mathematical surface model. If an urban road DEM is constructed on the basis of a previous road classification system, then the lateral morphological characteristics of the road will be seriously disregarded, resulting in the lack of road DEM fidelity and leading to errors in urban catchment analysis based on this urban road DEM. In addition, current urban road classification research focuses on the urban management perspective, particularly on the expression of road functions. By contrast, research on road classification and expression from the perspective of digital elevation modeling and that considers the morphological characteristics of urban roads is rare. Therefore, hierarchizing urban roads by considering their morphological characteristics and then finely constructing urban road DEMs are essential.

In this current study, we selected a typical urban road area in the Jianye district of Nanjing City in China as the study area, used the 1:500 DLG as the data source, and proposed a considering morphological characteristics fine (CMCF) method of urban road DEM construction. In particular, we aimed to (1) analyze the basic composition and morphological characteristics of urban roads, (2) hierarchize roads in accordance with different morphological characteristics from the perspective of DEM construction and then construct the corresponding DEMs, and (3) develop CMCF based on DLG data. Overall, the results of this study provide a basis and reference for complex urban road DEM construction.

## Materials and methods

### Study area

**Figure 1:**
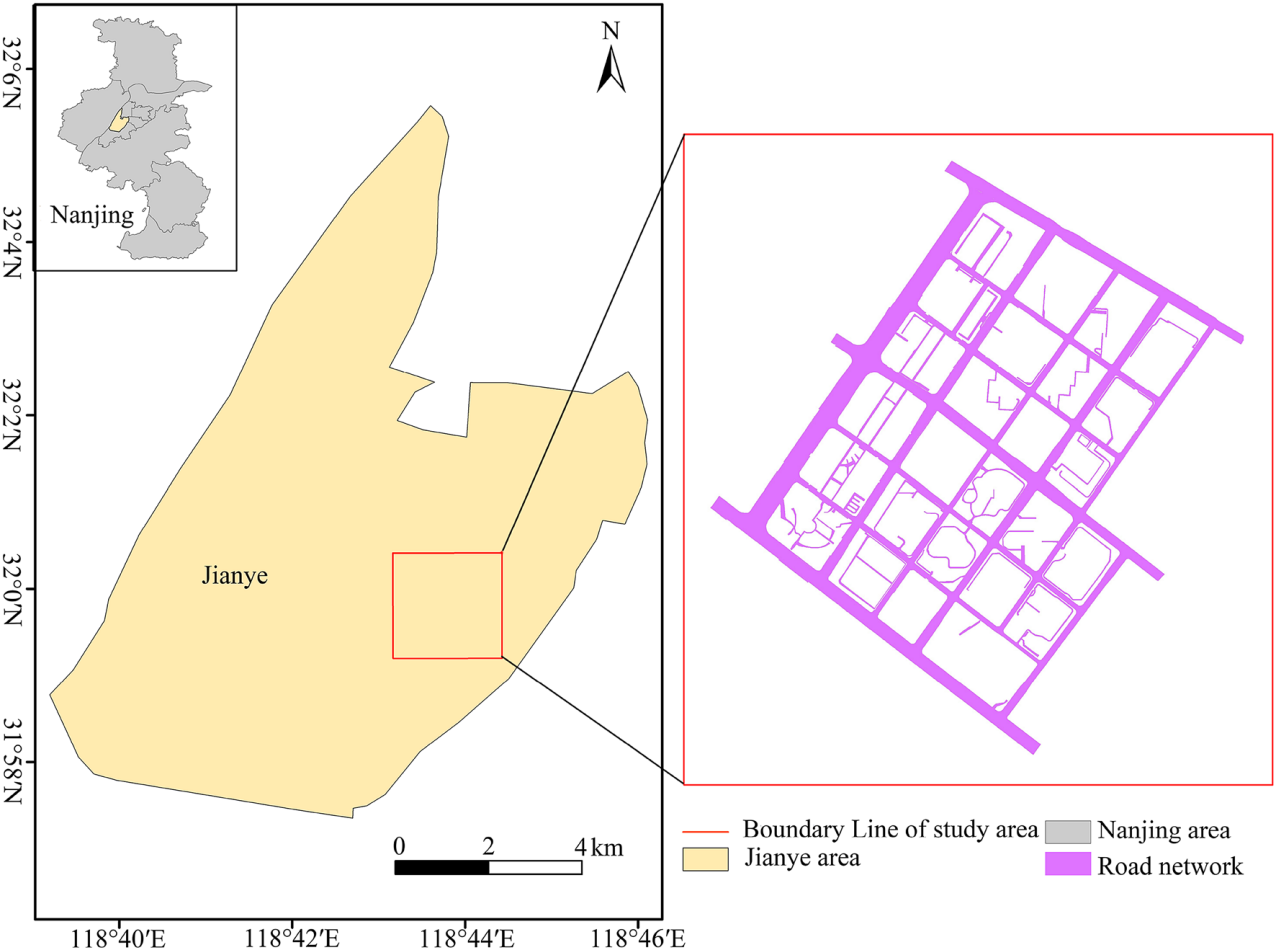
Location of the study area (Map of Nanjing from the China Administrative Divisions Map, review No.: GS(2019)1822, based on ArcMap v10.2 software, https://support.esri.com/zh-cn/Products/Desktop/arcgis-desktop/arcmap/10-2-2#downloads).

The current study focused on exploring a fine construction method for urban road DEMs. Therefore, the diversity and morphological characteristics of urban roads should not be the only factors to consider when selecting the experimental area, but also the availability of data for the area. To meet these requirements, a part of the central Jianye District of Nanjing City was selected as the study area (Fig. 1). The topography of the study area is significantly affected by human activities, the urban road network is complex, and the spatial elements are complete. Thus, the study area is ideal for investigating urban road DEM modeling.

### Data acquisition and preprocessing

Nanjing 1:500 DLG data was used as the experimental data source in this study. DLG data have a massive amount of urban road geographic information, including geometric, semantic, and elemental relationship characteristics. Thus, these data can provide accurate spatial location and attribute information for high-precision urban roads DEM construction, such as road centerlines, road edge lines, road surfaces, and elevation points. However, topographic line data in DLG data only include plane information without the corresponding elevation information, making the continuous spatial characteristics of urban roads difficult to describe. Consequently, the original elevation points cannot be applied directly to high-precision urban road DEM construction. In addition, different types of urban roads in DLG data have varied data collection processes in DEM construction, and thus, urban roads should be hierarchized in accordance with morphological characteristics. Therefore, performing a series of data pre-processing steps on DLG data, including urban road hierarchies, urban road information extraction and supplementation, and elimination of urban road elevation outliers, is necessary.

#### Urban road hierarchies

Previous urban road classification methods have focused on the expression of road level and utilization function without considering the morphological characteristics of roads. Thus, meeting the current increasingly high-precision and complex urban road DEM construction process is difficult. To express the lateral and longitudinal morphological characteristics of roads completely and restore the actuality of road DEM, the current study classifies urban roads into six categories in accordance with morphological characteristics: main lane, auxiliary lane, single lane, bicycle lane, sidewalk, and separation zone from the perspective of urban road DEM construction (Table 1).

**Table 1:** Morphological characteristics of urban roads.

Categories of urban roads	Morphological characteristics		Road level
	Lateral	Longitudinal	
Main lane	Continuous as a whole, with slightly elevation fluctuations	Single flat or curved surface	Level One
Auxiliary lane	Continuous as a whole and locally breakable, with slight elevation fluctuations	Single flat or curved surface	Level Two
Single lane	Overall gentle, with little elevation fluctuation	Single flat or curved surface	Level Three
Bicycle lane	Overall gentle, with a slightly undulating elevation at both ends	Single flat surface	Level Four
Sidewalk	Overall flat and discontinuous, with a slightly undulating elevation at both ends	Single flat surface	Level Five
Separation zone	Overall discontinuity, with undulating elevation	Non-single flat surface	Level Six

**Figure 2:**
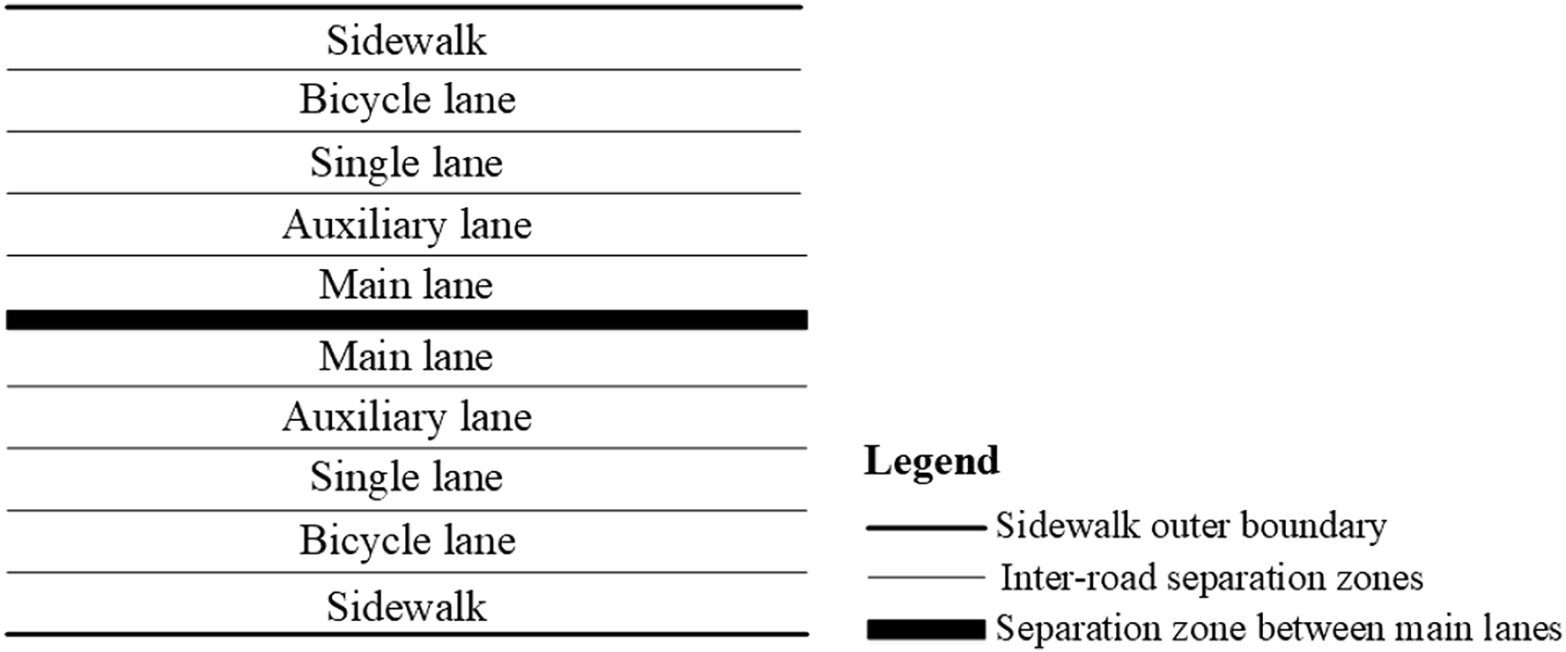
Schematic of the spatial combination of urban road elements under an ideal condition.

The spatial combination relationship of urban road elements under an ideal condition is illustrated in Fig. 2. From the road cross-section, the main lane is the road for motor vehicles, which is typically the core part of the road, located in the middle of the road, and the key element of road DEM construction. The auxiliary lane is generally located on both sides of the main lane, and it can be used by motor vehicles alone or shared by motor vehicles, electric vehicles, and bicycles, depending on planning needs. It is usually provided for vehicles that will make lane changes or turns. The single lane is located next to the auxiliary lane, and it can be used by bicycles, electric bicycles, and other nonmotorized vehicles. It usually includes the electric bicycle lane and part of the bicycle lane. In addition, separation zones can be appropriately added between the aforementioned categories of roads in accordance with the geometric width and functional needs of roads.

However, many different spatial combinations of urban road elements exist in practice due to the constraints of site and planning factors. The common combination forms are as follows: main lane + auxiliary lane + single lane + bicycle lane + sidewalk + separation zone, main lane + auxiliary lane + single lane + sidewalk + separation zone, main lane + auxiliary lane + sidewalk + separation zone, and main lane + single lane + sidewalk + separation zone. From the DEM modeling perspective, oriented to the multiple spatial combination changes of urban road elements, the main lane, auxiliary lane, single lane, bicycle lane, sidewalk, and separation zone are ranked from high to low in accordance with the level of road elements to divide them into one, two, three, four, five, and six levels, respectively (Table 1). That is, when a lower-level road is gradually integrated into a higher-level road in the extension direction, road morphology follows the principle that a higher-level road is superior to a lower-level road. The higher-level road is used as the DEM construction benchmark. For example, when the auxiliary lane joins the main lane, road DEM is constructed with the morphological characteristics of the main lane as the benchmark.

#### Extraction and supplementation of urban road information

In accordance with the urban road hierarchical method from the DEM perspective, the information of the main lane, auxiliary lane, single lane, bicycle lane, sidewalk, and separation zone in the study area is extracted separately from the DLG database. Subsequently, road surface, centerline, and corresponding elevation point data were extracted for each level of road elements on the basis of elevation, spatial, and semantic information.

**Figure 3:**
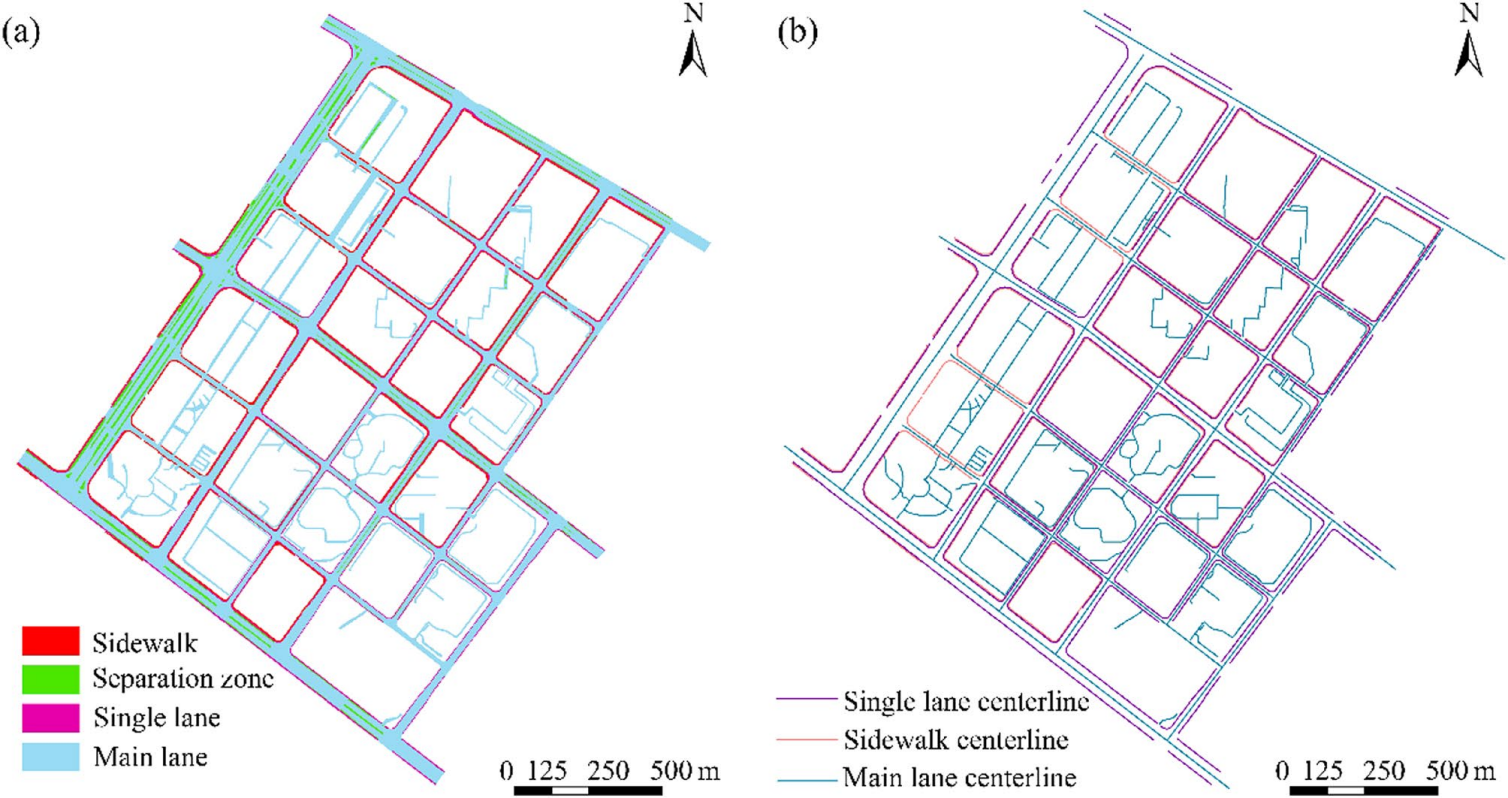
Urban road surface and urban road centerline after replotted.

The road surface of the main lane, auxiliary lane, single lane, bicycle lane, and sidewalk with a width greater than 1 m is replenished by using the DLG database of road edge line, road accessory facility surface, and residential surface data. In addition, although the DLG data include sufficient road surface and centerline data, problems, such as overlaps and voids at the junction of various road elements, occur. Therefore, checking the topology of each level of road is essential to ensure coherence and integrity between the individual road surfaces, particularly the core integrity of the main lane. Notably, road information is replotted on the basis of the longer main lane continuity when intersections are encountered. For the road surface after replotting and error correction, the new road centerline is re-extracted to produce complete road centerline data (Fig. 3).

#### Elimination of road elevation outliers

**Figure 4:**
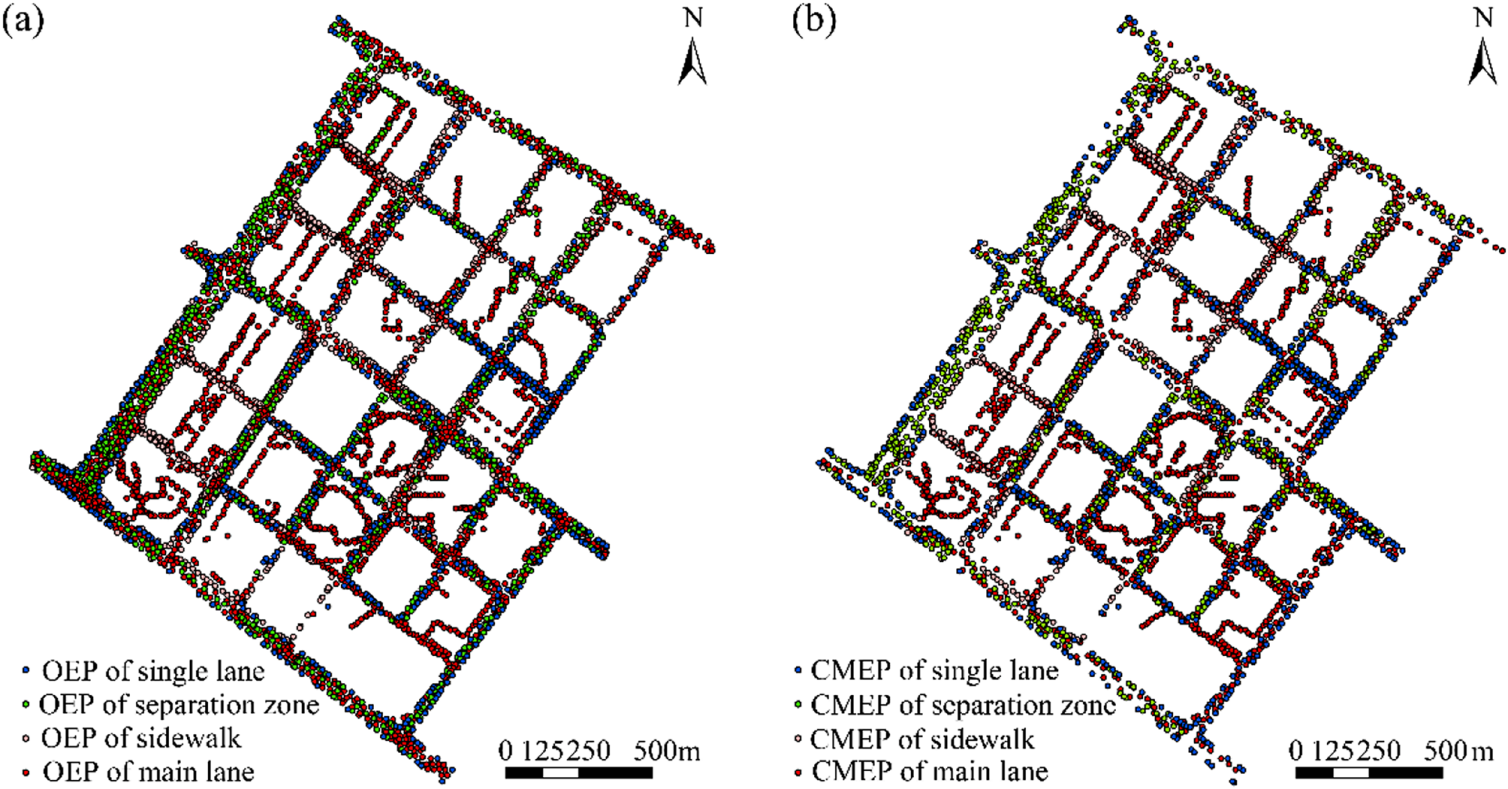
Comparison of urban road elevation outliers before (**a**) and after (**b**) their elimination.

As a result of various factors, such as measuring instruments, measuring process, and external environment, outliers inevitably occur in urban road elevation data. Urban elevation outliers are the abnormal abrupt change points within a road surface. They include global and local outliers, which are too high or too low than the whole trend surface of the road surface, destroying the morphology of urban roads and leading to the distortion of urban road DEMs. Therefore, eliminating the elevation point outliers of urban roads is particularly significant for urban road DEM construction. The elimination steps are as follows. In the first step, the exploratory spatial data analysis tool in ArcGIS software is used to initially search and eliminate road elevation outliers. In the second step, the remaining elevation points are constructed into a triangulated irregular network (TIN) and then transferred to a raster to produce a preliminary road DEM. Then, this road DEM is analyzed for mountain shadow rendering to eliminate elevation outlier data. In the third step, the improved Douglas–Peucker (DP) algorithm with an elevation change rate^30^ is applied to eliminate elevation outlier data that are inconsistent with road morphology. This step is repeated until no evident elevation outliers remain. In the final step, the elevation point data that meet the requirements are combined as the final urban road DEM construction elevation point data. The comparison of the original elevation points (OEP) of the urban roads and the constructed model elevation points (CMEP) with the elimination of outliers is depicted in Fig. 4.

### Urban road DEM construction method

The morphological characteristics (including longitudinal and transverse morphological characteristics) of different levels of urban roads vary significantly; thus, hierarchically constructing DEMs of different levels of urban roads is necessary.

#### Construction method for the main lane

**Figure 5:**
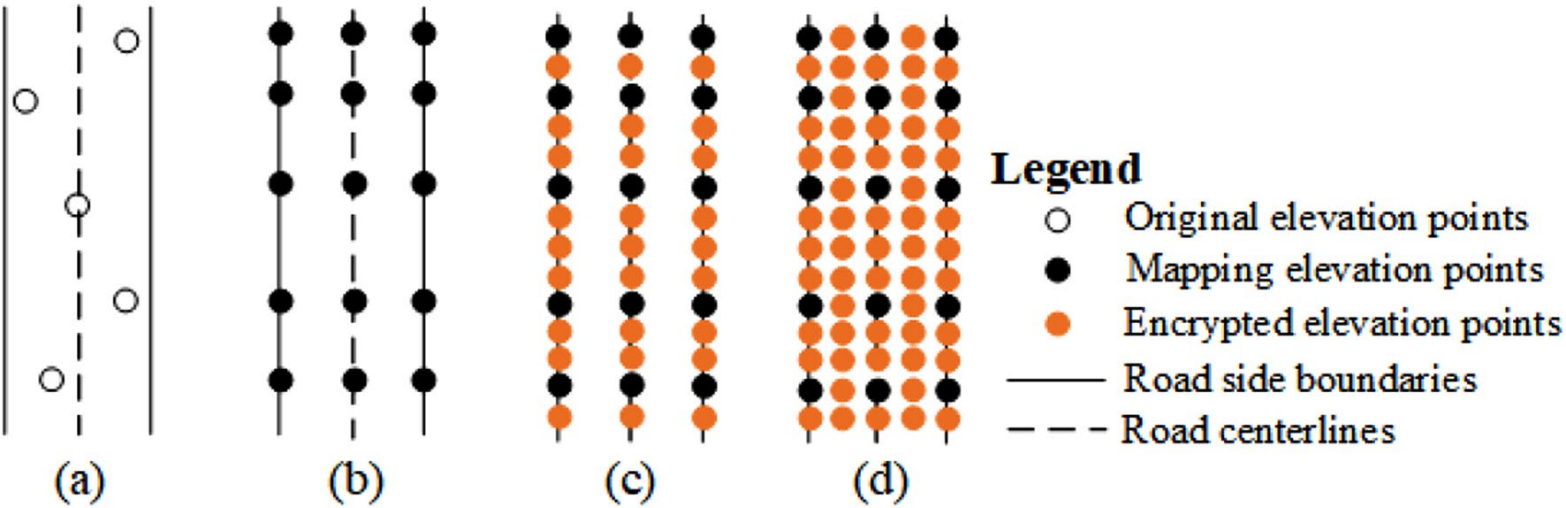
Main lane DEM construction method.

The main lane is the core spatial element of the whole urban road network, and an accurate representation of the main lane model provides an important foundation and support to urban road DEM construction. The following steps are used to model the main lane (Fig. 5).

1. *Elevation points assignment mapping* The extracted main lane elevation points are not strictly located on the road sideline, as shown in Fig. 5a. Thus, mapping the extracted road elevation points vertically to the main lane sideline (generated by the main lane road surface) and main lane centerline, as depicted in Fig. 5b, is necessary.
2. *Elevation point encryption* The density of topographic elevation points in DLG data is relatively sparse and insufficient to support high-precision urban road DEM construction. According to the industry standard of “Digital products of fundamental geographic information 1:500 1:1000 1:2000 digital elevation models” (i.e., 1:500 high-precision urban DEM grid size is 0.5 m)^39^, we use the equal spacing encryption method to encrypt the mapped elevation points at equal spacing of 0.5 m from the longitudinal section (Fig. 5c) and cross section (Fig. 5d) directions.
3. *Main lane DEM construction* The main lane DEM is produced via inverse distance weighting (IDW) on the basis of uniformly distributed elevation points, road centerlines, and urban road surface data after encrypted interpolation.

#### Auxiliary lane and single lane construction methods

The auxiliary lane and single lane have the same morphology as the main lane in urban roads under most situations, and their construction approach is consistent with that of the main lane. However, some morphological characteristics at the junction of the auxiliary lane and single lane with the main lane do not match the significant height difference of the main lane, which is inconsistent with the actual gradual integration of roads of all levels.

Therefore, to overcome the problem that the end of the road cannot be smoothly transitioned in the DEM construction of the auxiliary lane and single lane, the elevation values at the intersection of the end centerline and the road surface are extracted from the main lane DEM and involved in the classification modeling of the auxiliary lane and single lane to control road end morphology, and finally, construct auxiliary lane DEM and single lane DEM.

#### Bicycle lane and sidewalk construction methods

The lateral morphological characteristics of the bicycle lane and sidewalk are single flat and straight surfaces, and the construction of the bicycle lane DEM model can refer to the construction method of the main lane DEM. The construction of the sidewalk road DEM model can be divided into four situations in accordance with the number of elevation points.

1. *Sufficient road elevation points* The sidewalk DEM construction method is the same as the aforementioned main lane DEM construction method.
2. Insufficient road elevation points and road surface has only one elevation point. A sidewalk generally exists in blocks, and its longitudinal length is shorter than those of other road levels. Longitudinal elevation exhibits nearly no change. A plane can be determined by one elevation point to express the surface of the sidewalk.
3. Road elevation points are insufficient, and road surface has only two elevation points. If the two elevation points are located at the beginning and end of the road, then these points can be used to determine a short flat straight plane to express the surface of the sidewalk. If the two points are not strictly located at the beginning and end of the road, then they can be handled in accordance with the second situation, i.e., find the average to determine the sidewalk road surface.
4. No elevation points on the sidewalk road surface. No model is constructed for this situation.

#### Separation zone construction method

Separation zone morphology is evidently different from the other levels of road morphology; it is essentially a road boundary at all levels. Whether the separation zone can be constructed in urban road DEM depends on the effect of its shape or width. In general, only the non-cutaway type of separation zones with a width greater than 1 m can be constructed in urban road DEM. Then, the actual local morphology of the separation zone is constructed in the model by using the corresponding construction method, such as the traditional TIN construction method to build a natural undulating separation zone and the flat surface method to construct an artificially modified separation zone.

Similarly, the distribution of elevation points is uneven in the separation zone surface, and some of the separation zones have a sufficient number of elevation points for normally constructing the model, while others have fewer elevation points and unable to build the model. To address the aforementioned problems, elevation points within the non-urban road surface, i.e., elevation points of non-urban buildings, flat surfaces, slopes, and other special feature elements in urban parcels, and the road boundary line, steep hill line, and slope line elements are used as constraints to automatically encrypt the remaining elevation points for constructing DEM^28^, and finally, the separation zone model is clipped based on the boundary line of the separation zone.

#### Merging of models

The aforementioned hierarchically constructed urban road DEMs are merged to generate the complete urban road DEM. In accordance with the core construction principle of the main lane, the main lane DEM is extended to the single lane or bicycle lane, and then the auxiliary lane DEM is binarized and embedded into the main lane extended surface model by using the conditional function tool to obtain the “main lane + auxiliary lane” DEM. The preceding mosaic method is repeated to embed the single lane, bicycle lane, and sidewalk DEMs into the previous mosaic result to obtain the “main lane + auxiliary lane + single lane”, “main lane + auxiliary lane + single lane + bike lane”, and “main lane + auxiliary lane + single lane + bike lane + sidewalk” DEMs. Finally, the separation zone DEM is embedded into the “main lane + auxiliary lane + single lane + bike lane + sidewalk” DEM in accordance with the same method to complete all the merged models. Road surface buffer analysis is performed to eliminate the problem of jagged gaps that occur at different levels of urban roads during the merging of models. In the end, topological checks are used to ensure the correctness of the spatial relationships of all levels of urban road DEM. In addition, slope data are extracted from the constructed urban road DEM for further analysis.

## Results

### DEM construction results for different road levels

**Figure 6:**
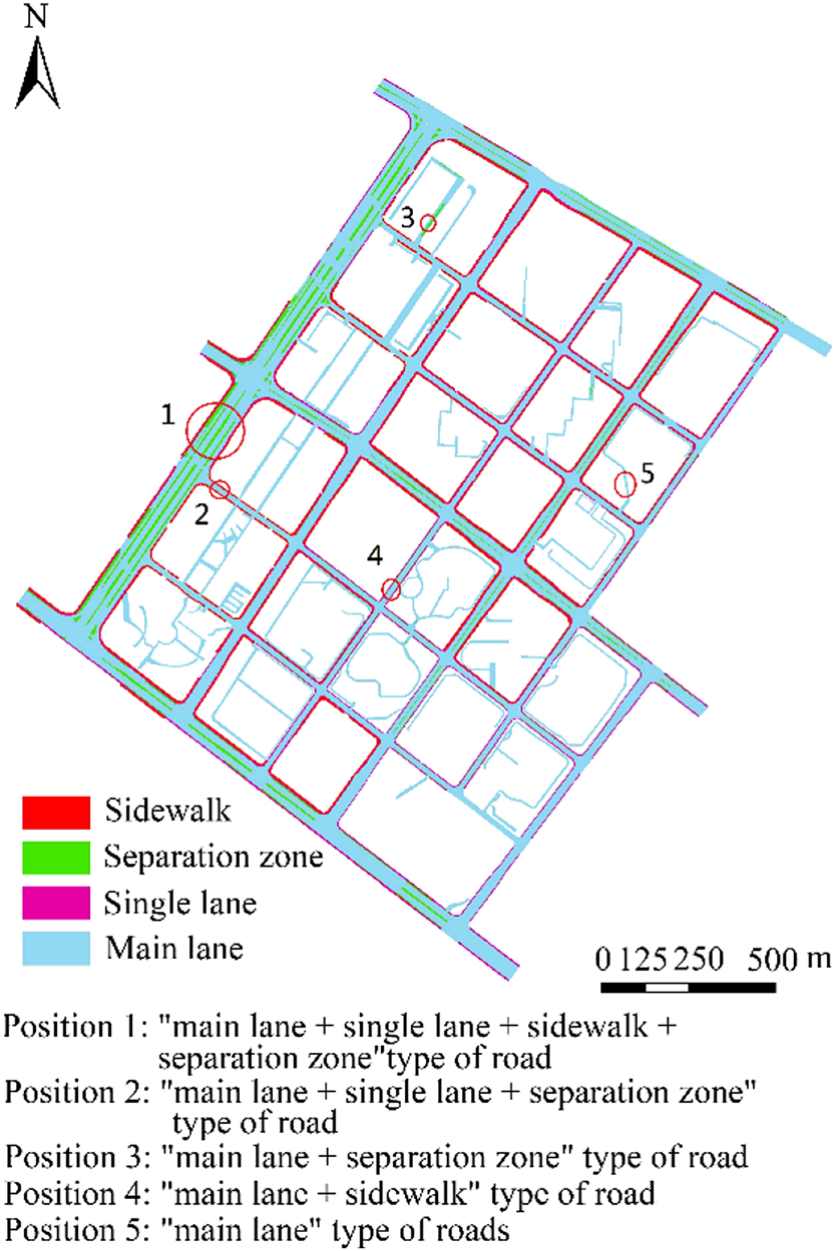
Result of urban road hierarchy.

**Figure 7:**
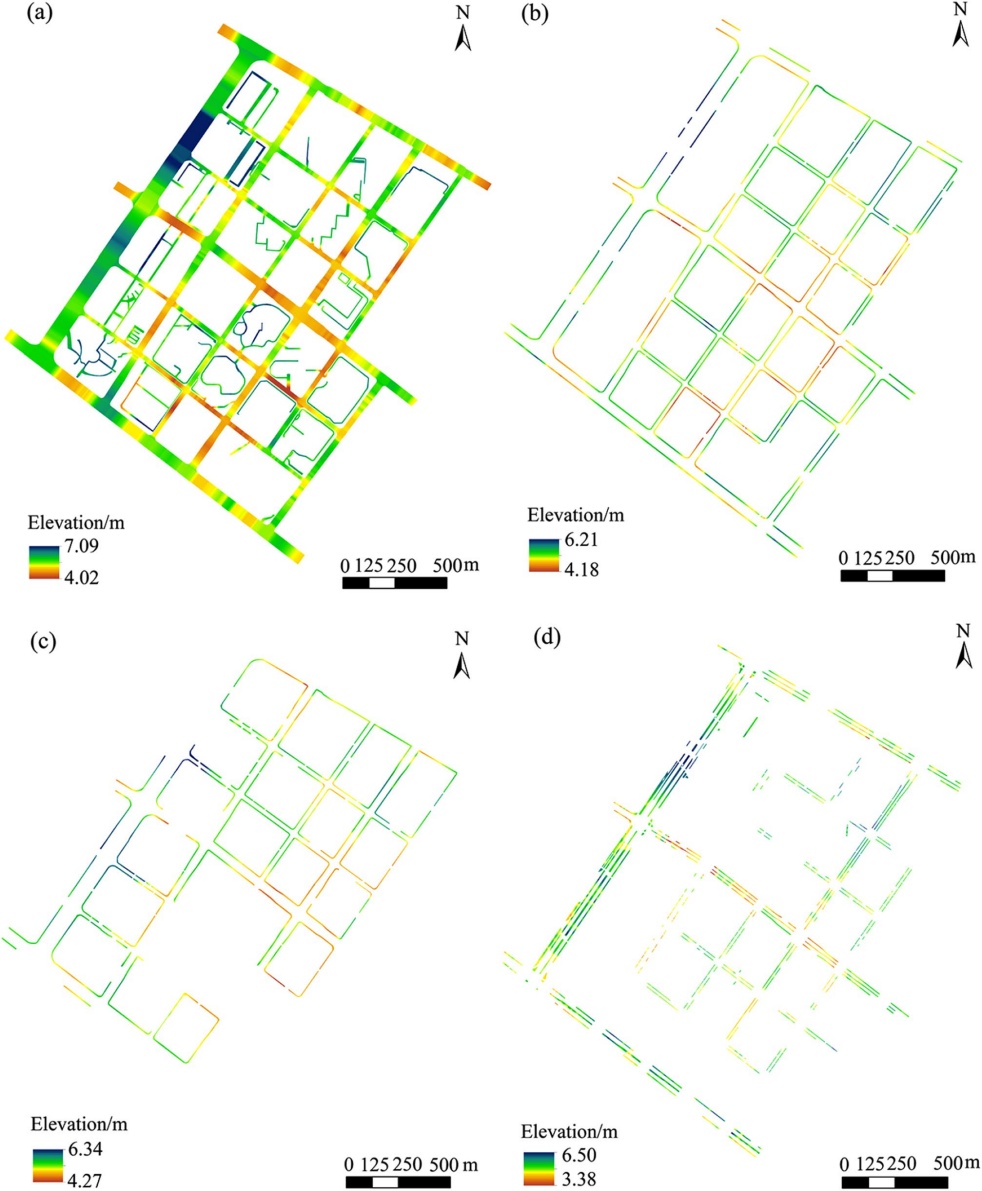
DEM construction results for different levels of urban roads.

**Figure 8:**
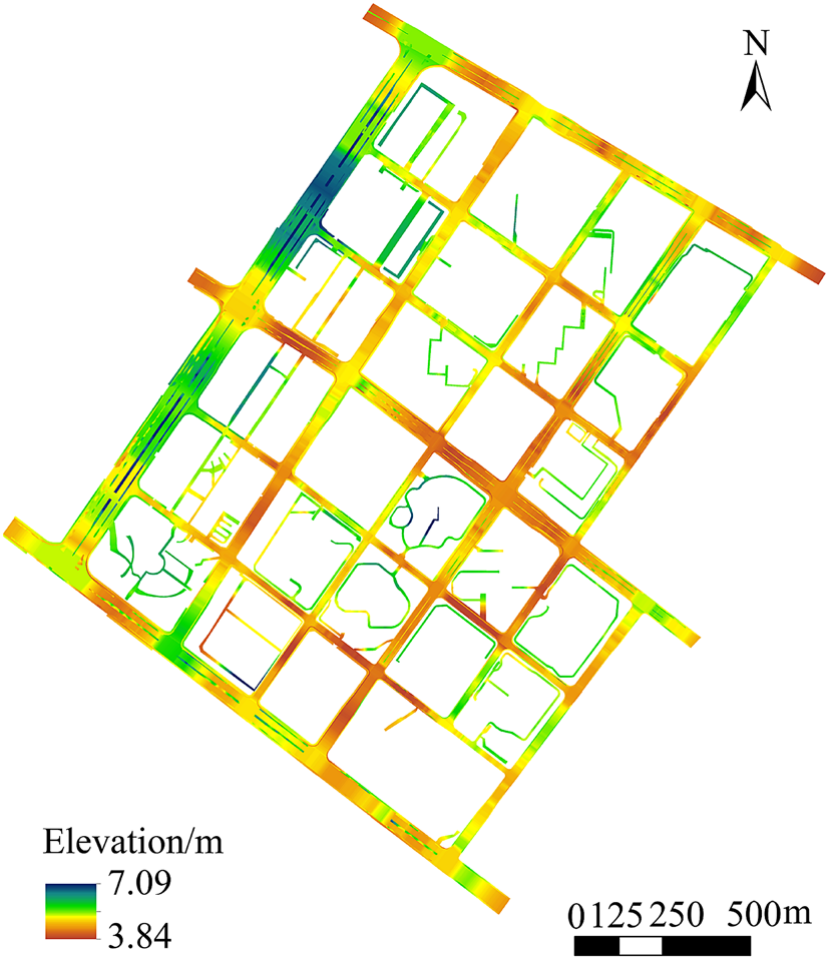
Merged urban road DEM model.

In accordance with the aforementioned different levels of urban road DEM construction methods, the DEM construction results of urban roads in the study area are presented in Fig. 7. Given the lack of auxiliary lane and bicycle lane in the study area, these levels of urban roads are not discussed below. The primary purpose of the current study is to develop a fine construction method for the DEM of urban roads that considers morphological characteristics. Nevertheless, the DEM construction methods of auxiliary roads and bicycle paths in other areas can still refer to the corresponding construction methods proposed in this study. The research team of the authors has already implemented a high-precision urban road DEM construction, including auxiliary lane and bicycle lane, in other areas of Nanjing on the basis of the method proposed in this work. As shown in Fig. 6, the different levels of urban roads have clear road boundaries and relatively complete expression of morphological characteristics, meeting the requirements of high-precision urban road DEM construction. The DEM construction results of different levels of urban roads and the merged DEMs for each level of urban roads are presented in Figs. 7 and 8, respectively.

As illustrated in Fig. 8, the merged urban road DEM can clearly present the boundaries of different levels of urban roads and preserve well and express the real morphological characteristics of urban roads. A conclusion can be drawn that urban road DEM constructed using the CMCF method proposed in this work can completely express the morphological characteristics of urban roads and the boundary information of all levels of roads.

### Accuracy assessment

The urban road DEM constructed using the CMCF method of urban road DEM is compared with the models constructed using previous construction methods (including direct and indirect methods), and the accuracy of the constructed DEM is evaluated in terms of elevation accuracy. Direct methods are used to directly construct the regular grid urban road DEM via the IDW method. The indirect methods are based on urban road elevation point data, which are used to construct a TIN model that continuously covers the whole study area. Then, the TIN model is converted into a regular grid raster urban road DEM. Notably, the data used to construct the urban road DEM models by using the IDW and TIN methods eliminated urban road elevation outliers.

In this work, a cross-validation method is used to evaluate the accuracy of the model based on two evaluation indicators: the mean error (ME) and the root-mean-square error (RMSE) (Table 2). In the cross-validation method, 20% of the total elevation points are selected for elevation accuracy validation. The accuracy evaluation results show that the ME and RMSE of the urban road DEM model constructed on the basis of the CMCF method are smaller than those of the IDW and TIN methods, with ME and RMSE of 0.015 and 0.060, respectively; model accuracy is higher and satisfies the Level 1 flatland standard of urban road DEM construction^39^.

**Table 2:** Accuracy assessment results.

Methods	ME	RMSE
IDW	0.146	0.207
TIN	0.149	0.211
CMCF	0.015	0.060

### Comparison between urban road DEMs constructed using the CMCF method and previous methods

**Figure 9:**
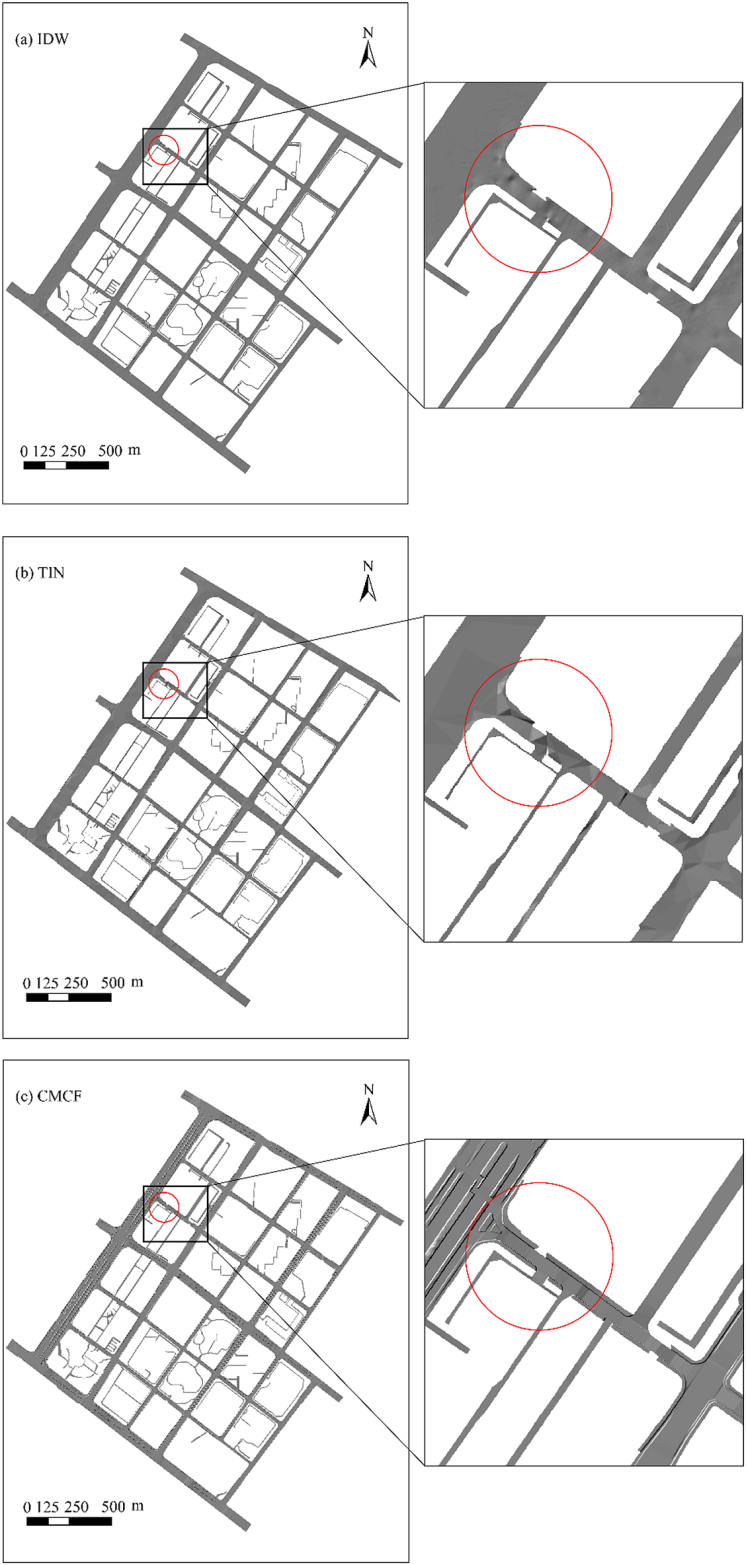
Comparison of the results of constructed models.

As illustrated in Fig. 9a,b, the urban road DEMs constructed using the IDW and TIN methods exhibit a certain degree of road mutation distortion, and the constructed roads have large undulations and rough and uneven road surfaces. The realistic road surface should be a flat surface and generally without large bumps. In addition, the road DEM constructed using the IDW and TIN methods only roughly expresses the overall morphology of the road. In the cross-section, some sections have large slope undulations, which do not accurately express the cross-sectional characteristics of urban roads. In the longitudinal direction, although the road as a whole is continuous, the road abruptly changes in local areas, resulting in the serious distortion of road morphology in some sections. Meanwhile, the two methods also disregard the spatial element combination level of the road cross-section, lack consideration of road morphology, and the constructed model provide poor results.

**Figure 10:**
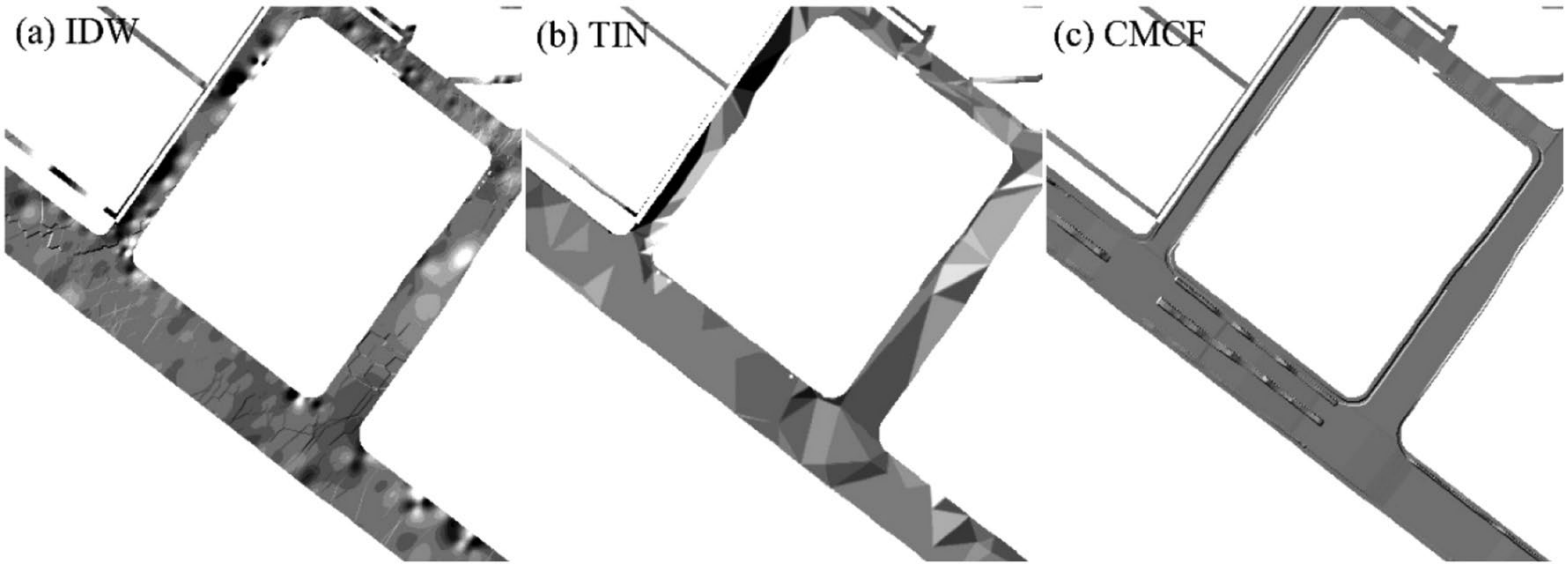
Intersection results of different construction methods.

By comparison, the DEM of urban roads constructed using the CMCF method can more completely and accurately express the morphological characteristics of roads. Moreover, the model provides better results (Fig. 9c). In particular, the urban road DEM model constructed based on the CMCF method can accurately express the morphological characteristics and hierarchical relationships of all road levels. Meanwhile, the model exhibits small elevation changes of all levels of road elements in the lateral direction, the flat surface characteristics are better expressed, and the abrupt variability characteristics of sidewalk and separation zone road boundaries are consistent with the actual morphology. In the longitudinal direction, the characteristics of the continuous high and low undulations of urban roads are expressed, and no evident abrupt change in road localization is observed. Meanwhile, the urban road DEM model constructed using the CMCF method effectively avoids the problem of abrupt changes in elevation at the main lane road connections in the models constructed using the IDW and TIN methods; it also better enhances smoothness between intersections (Fig. 10).

**Figure 11:**
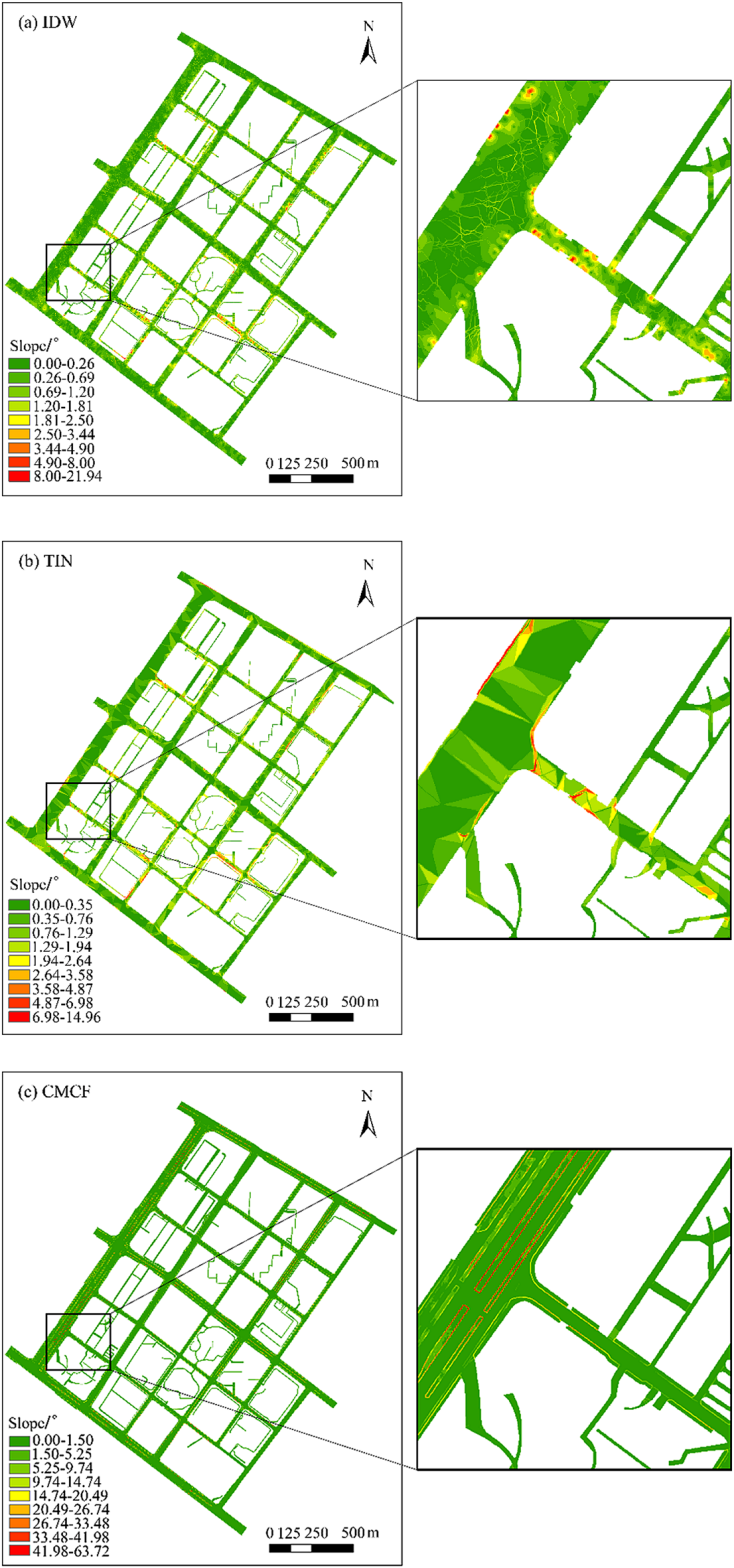
Road slope maps extracted using different model construction methods.

In addition, a certain height difference generally exists between the main lane and the sidewalk, and it cannot be accurately expressed by the models constructed using the IDW and TIN methods. By contrast, the model constructed using the CMCF method can effectively express this topographic characteristic, which is more realistic, reliable, and in line with the actual situation. By comparing the road slope maps extracted from the DEMs constructed using different methods (Fig. 11), significant slope variations can be observed on the road surface of each road level in the road DEMs constructed using the IDW and TIN methods, particularly at the connection of various road levels, which is clearly contrary to the actual situation (Fig. 11a,b). By contrast, a larger slope difference is observed between different road levels in the urban road DEM constructed using the CMCF method, and the slope remains flat within each individual road level, which is consistent with the semantic characteristics of a smooth and gradual change in road. Furthermore, the road slope map extracted from the urban road DEM constructed using the CMCF method can intuitively reflect that the CMCF method effectively addresses the problem of smooth transition from the end of the single lane to the main lane. Moreover, no evident high bump occurs at the end of the road, better corresponding to the actual road morphology (Fig. 11c).

For the separated zone, the model constructed using the CMCF method can clearly express its boundary contour and quickly distinguish it from other levels of urban roads. For the single lane, no evident high bump occurs between the road end and the main lane, enabling good smooth transition connection. By contrast, the urban road DEMs constructed using the IDW and TIN methods exhibit large variations in road slope and a large number of abrupt topographies on the road surface. They also cannot distinguish different road levels because the two methods regard the different morphological characteristics of roads as a whole when constructing a model. Overall, the urban road DEM constructed using the CMCF method proposed in this study can more finely and accurately express the morphological characteristics of urban roads and better restore the surface morphology of actual roads.

## Discussion

In this study, we proposed the CMCF method for urban road DEMs from the perspective of DEM construction. The method used large-scale DLG data as data source, analyzed the basic constituent elements of urban roads and variable urban road element combination forms, hierarchized urban roads with different morphological characteristics, constructed the corresponding DEMs, and finally merged the hierarchical road DEMs into the final urban road DEM. Two evaluation indicators, i.e., ME and RMSE, were used to assess the accuracy of the model. Compared with previous DEM construction methods (e.g., the IDW and TIN methods), the urban road DEM constructed using the CMCF method exhibited higher accuracy and could accurately express the morphological characteristics of different road levels, meeting the Level 1 flatland standard for urban road DEM construction.

However, the urban road DEM constructed using the CMCF method has some limitations. Urban large-scale DLG data will inevitably produce measurement errors in topographic mapping, such as elevation errors of control points and spatial relationships are not strictly matched, due to the influence of various factors. Although we eliminated road elevation outliers before constructing an urban road DEM, the errors in the data source were difficult to eliminate completely, affecting the results of constructing the model to a certain extent^7^. Due to the data structure limitation of the grid data, the pure grid road DEM experiences difficulty in expressing more detailed road abrupt terrain, such as road curbs, sidewalk steps, and separation zone curbs; thus, introducing F-DEM^40^ or other vector-grid integrated DEM methods is necessary to construct the corresponding models. Only DLG data are used to construct the urban road DEM in this work. Multisource data may provide richer morphological characteristics for urban roads, improving the results of urban road DEM construction^41,42^. 3D oblique photographs that contain elevation and image information^43,44^ and open-source street view data with rich fine geographic scene information^45,46^ can be used as data sources for urban road DEM construction. These data will strongly complement the local morphological characteristics of urban roads. In addition, we will increase our effort to determine how to fuse multisource data and develop more optimal classification interpolation algorithms for constructing urban road DEMs in our future work.

Despite the preceding limitations, the urban road DEM constructed using our proposed CMCF method more accurately expressed the morphological characteristics of all levels of urban roads, and the accuracy of the model met Level 1 flatland standard for urban road DEM construction. This study can provide a new method for urban road DEM construction and data support for urban 3D visualization and urban surface rainfall flood simulation.

## Conclusion

In this study, we proposed an urban road DEM construction method that considered the morphological characteristics of urban roads (i.e., the CMCF method) from the perspective of DEM modeling in response to the shortcomings of previous urban road classification methods that focused on the expression of road functions and lacked consideration of road morphology. These methods could not accurately express the morphological characteristics of urban roads and could not meet the needs of high-precision urban road DEM construction. The proposed method analyzed the basic constituents of urban roads and the combination of variable urban road elements, summarized the morphological characteristics of six levels of urban roads (e.g., main lane, auxiliary lane, and single lane), and provided a theoretical basis for future urban road data extraction and DEM construction.

The CMCF method formed an integrated “hierarchical–acquisition–interpolation–construction–merging” urban road DEM construction method. It adopted different construction methods for the main lane, auxiliary lane, single lane, bicycle lane, sidewalk, and separation zone, particularly in the model construction method for the main lane. It established the construction technique of “elevation point allocation mapping–elevation point encryption–elevation point interpolation–DEM construction for the main lane”. In addition, the road DEM constructed using the CMCF method conformed to the road semantic characteristics of the city on a macro scale, and no abrupt change of local road surface abnormalities was observed, with a clear framework structure of urban road elements on the micro-scale. This finding is consistent with actual urban road morphological characteristics. Overall, the urban road DEM constructed using the CMCF method can more accurately express the morphological characteristics of all levels of urban roads. It achieves higher model accuracy than the previous DEM construction methods (e.g., the IDW and TIN methods), meeting the Level 1 flatland standard of urban DEMs and providing new insights into the construction of high-precision urban road DEMs.

## Data Availability

The datasets generated and/or analysed during the current study are not publicly available due to privacy restrictions but are available from the corresponding author on reasonable request.
